# Editorial: Plant responses to environmental stresses based on physiological and functional ecology

**DOI:** 10.3389/fpls.2023.1290405

**Published:** 2023-10-11

**Authors:** Kaixiong Xing, Hongbo Li, Deliang Kong, Chen Chen

**Affiliations:** ^1^ School of Life Science, Hainan Normal University, Haikou, China; ^2^ Institute of Environment and Sustainable Development in Agriculture, Chinese Academy of Agricultural Sciences (CAAS), Beijing, China; ^3^ College of Forestry, Henan Agricultural University, Zhengzhou, China; ^4^ PostDoc Position of Laval University, Quebec City, QC, Canada

**Keywords:** plant physiological processes, plant functional traits, environmental stresses, climate changes, functional ecology

Plants require a proper balance of matter and energy to maintain their survival and reproduction. Biotic and abiotic stresses in diverse environments can influence plant photosynthesis, water and nutrient acquisition, and utilization. Plant functional traits play a crucial role in functional ecology as they constitute the fundamental elements and critical links between plant physiology and plant function. Although applications of plant functional traits research have been diverse, there are several novel areas where research on plant functional traits can significantly contribute in the future. The study of plant physiological and functional ecology has developed a comprehensive understanding of the responses of individual plant traits and the integration of plant responses to environmental change. Despite the increasing number of studies attempting to establish a linkage between plant physiological processes and functional traits, these covariations have received limited theoretical and experimental verification. We propose this Research Topic to bridge the gap between physiological processes and functional traits in plants. Several meaningful discussions have emerged from this compilation, shedding light on plant responses to environmental stresses and the underlying mechanisms. Research on the topic has even extended into several novel directions, such as the study of heavy metal and organic pollutant responses, the role of microplastics, and the impact of fungi on plant physiology.

Understanding the relationship between plants and the climate is necessary to make predictions across trophic levels. The prevalence of herbivores is associated with variations in plant functional traits, including physical and chemical defenses. In the context of global climate change, research on plant defense responses to herbivory stress should not only distinguish direct changes in plant traits resulting from biotic factors but also consider the multilayered canopy functional structure of communities modified by the introduction of herbivores (Alexander et al., 2018). Besides the direct effects of climate change (Gilman et al., 2010), colonization by novel herbivores in alpine ecosystems (presumably due to warming) has resulted in a rapid reorganization of plant community structure and traits, adding new and complex layers to how we imagine ecosystems might respond to climate change (König et al., 2022). These results underscore the complex dynamics between plants and herbivores that complicate our understanding of how plant physiology and function may change in the climate of the Anthropocene. For example, an experiment on greenhouse warming in alpine meadows in Switzerland found that herbivore incursion in warming treatment plots significantly decreased the vegetation biomass of the entire plant communities and changed the functional dominance of the community-weighted mean traits (Descombes et al., 2020).

Based on enough field investigations and manipulative experiments, plant ecological stoichiometric traits are tightly correlated with plant functional traits and environmental factors. Research on plant nitrogen and phosphorus contents and nitrogen-phosphorus stoichiometric relationship are often the focus of plant ecologists. However, increasing focus has been paid to the role that other elements, such as drought resistance (related to potassium), light environment (related to magnesium), herbivory and disease resistance (related to silicon) (El-Shetehy et al., 2021; Johnson et al., 2021), and resource allocation (related to calcium) (Xing et al., 2021), play roles in regulating plant communities. In general, this approach has led to the identification of several ‘limiting’ nutrients by which plant species distribution is governed (Han et al., 2011). This principal – first explained in the 19th century by the German scientist Justus von Liebig, is often referred to as the “Law of the Minimum.” (Sterner and Elser, 2003), Increasing work looking into the relationship between plants and their abiotic environment has confirmed this principal in a number (4149) of forest communities in China (Tang et al., 2018). This rule allows for the development of biogeochemical ‘niches’ by the geographic or temporal distribution of species that are limited by the distribution of their nutrients (Peñuelas et al., 2019). These niches are often used as a tool to detect responses in plant communities to environmental change. For example, suppose a given species of plant requires a certain optimum range of soil nutrient and pH conditions (an environmental niche). We can observe how the demography of this plant is influenced by a changing climate (such as by aerosol deposition of nitrogen) as the climate influences the soil availability of nutrients and pH. To be clear, studies on the chemical stoichiometric characteristics of plant elements (except carbon, nitrogen, and phosphorus) are still lacking. Although large-scale patterns of plant stoichiometry traits have been reported in a few studies, the formation and driving mechanisms of their variation are not clear.

We mention the context of climate change given its immediate relevance when we consider how we understand plant physiology and function. Environmental change can increase the frequency and severity of stresses, including flooding, drought, salinity, freezing, species migration and innovation, human interference, and new types of pollution such as microplastics and nuclear pollution. The exploration of plant diversity (in both physiology and function) within and between species can help understand which trade-offs reflect the adaptation mechanisms of plants to environmental stress and which combinations of traits may pre-mitigate environmental stress. A clear definition of plant stress resistance remains a primary challenge, and resolving this problem will greatly facilitate the analysis of plant genetic and phenotypic properties and stress resistance through laboratory experiments and field observation methods (Verslues et al., 2023).

Future research in this area can make significant contributions, such as improving human health through food nutrition and quality. However, there are several aspects that should be addressed or further emphasized in future research, including the development of conceptual frameworks and models linking physiological processes and functional traits, the investigation of abiotic and biotic stresses, the study of plant nonlinear and comprehensive responses to multifactorial stresses, case studies focusing on specific nutrients, components, and organs, and the examination of plant physiological process changes and functional trait variations in ecological restoration and succession (Johns et al., 2022; Yang et al., 2023). In conclusion, the study of plant physiological processes and functional traits is crucial for understanding plant responses to environmental stresses. This Research Topic has provided valuable insights into the linkages between physiological processes and functional traits, but there are still many unresolved questions and areas for future research. By addressing these gaps, we can further enhance our understanding of plant responses to environmental changes and contribute to various fields, including ecological restoration, food nutrition, and human health.

The case studies, hypothesis and theory articles published in this Research Topic provide additional insights into plant responses to environmental stresses based on physiological and functional ecology. The focus on trait-based approaches is designed to arm researchers and policy makers with quantitative measures of plant responses to environmental stress. Consequently, we would like to focus on direct measurements of new physiological and phenotypic traits that may be related to plant functions, particularly in scenarios where our potential for data collection has significantly improved over recent years. These new techniques and projects quantifying the mechanistic responses of plants to environmental change allow for the development of better models and predictions of vegetation change in a changing climate. Thus, a priority for future studies is to establish better links between precise organismal traits and plant functions, particularly on the analyses of plant traits related to newly human-induced environmental changes, such as increased microplastics, inevitable nuclear-contaminated water pollution, increased invasive species, more frequent climate anomaly and so on.

## Author contributions

KX: Writing – original draft. HL: Writing – review & editing. DK: Writing – review & editing. CC: Writing – review & editing.
